# Creating welcoming and courageous spaces for student leadership training

**DOI:** 10.1002/yd.20646

**Published:** 2024-11-28

**Authors:** Darquillius J. Mayweather

**Affiliations:** ^1^ Watson College of Education University of North Carolina Wilmington Wilmington North Carolina USA

## Abstract

The evolving demographic landscape in the United States is a cause to shift leadership training approaches to better serve diverse populations. This article addresses the call for leadership educators to create inclusive, courageous training environments that reflect the increasing diversity in educational and professional settings. Traditionally, leadership education has centered on White and masculine paradigms, often neglecting the unique experiences of historically marginalized groups. This article emphasizes the importance of recognizing and incorporating social identities into leadership training by using these four key strategies: (1) building trust, (2) acknowledging social identity, (3) identity‐based storytelling, and (4) experiential learning activities. By integrating these strategies, leadership educators can foster environments that are conducive to deeper learning and engagement.

## INTRODUCTION

In the coming decades, the demographic makeup of our training experiences will change. The population of White Americans will decrease to 47%, compared to 67% in 2008 (Passel & Cohn, 2008). In education, the White student population is decreasing, students from racially and ethnically underrepresented groups are increasing, women attend college more than men, nontraditional age and experience students are growing, and the need to understand gender identity and expression is expanding (de Bray et al., 2019; Harbin, 2016; Parker, 2021; Schwartz, 2023). Leadership educators must be prepared to create courageous and welcoming environments for participants from various underrepresented identities and backgrounds. This is a call to make leadership training more culturally relevant (Jones et al., 2016). Ignoring the demographic changes in America and American higher education has serious implications for leadership educators, participant satisfaction, participant experiences in training, and participant's practice beyond training. Conventional leadership training regularly fails to include historically underrepresented and disadvantaged populations (Kezar & Lester, 2010). This article will cover how trainers can create welcoming and courageous environments across social identities by (1) building trust, (2) acknowledging social identity, (3) identity‐based storytelling, and (4) experiential learning activities.

Leadership research traditionally focused on White and masculine‐centered concepts until researchers began focusing on race and gender, which ushered in contemporary leadership research to include historically marginalized social groups (Dugan, 2017; Kezar & Lester, 2010). Emerging cross‐cultural leadership theories moved leadership educators past leadership identity just as personality types, values, and labels towards recognizing social identity and sociocultural experiences that shape one's practice and praxis (Haslam et al., 2017). Social identity is complex, multidimensional, fluid, in motion, and ever‐evolving (Jones & Abes, 2013; Jones et al., 2016); it accounts for how individual and group advantages and disadvantages shift depending on the location, context, and systems of oppression (Adams et al., 2007). As emerging leadership researchers acknowledge social identity and positionality, leadership educators need identity‐based leadership training guidance and pedagogy to enhance their facilitation skills in diverse training contexts.

### Positionality

As a qualitative researcher, I believe social identity provides a window into stories people tell about their lived experiences. Stories help individuals, groups, and communities understand a person's positionality in society (Brown, 2018). In Mahoney's (2017) article on culturally relevant leadership, the author discusses how positionality shapes our being and informs our responses in society. Positionality is shaped by social identity, societal perspectives on social identity, and individual and collective lived experiences within society. A person's positionality through a lens of intersecting social identities is inextricably connected, complex, fluid, contextually bound, and associated with power (Kezar & Lester, 2010). Leadership educators should constantly assess their positionality and social identity memberships as they work to foster connection and learning in training (Kroll, 2023).

As a heterosexual, able‐bodied male who is married, a father of two able‐bodied children, a PhD‐holder, and a homeowner, I hold multiple privileged identities. These identities simultaneously operate in conjunction with multiple marginalized or underrepresented identities I hold, including being Black, a first‐generation college student, raised in a working‐class single‐parent home, public school educated (in a high school that predominantly produced factory workers), first‐generation management‐level professional, and living with mental wellness issues. Leadership educators can use their identities as meaning‐making tools to create a welcoming, courageous training space to increase vulnerability, empathy, and, ultimately, learning. My training journey started when I taught an intergroup relations course early in my career. Until then, I never took the time to understand how social identities and their intersections impacted student leadership learning, practice, and praxis. Taking responsibility for the diversity training of students as a student affairs practitioner transformed me into a leadership educator.

This article is for leadership educators across sectors of the educational system within the United States. If you are employed or have been employed to train youth, young adults, or emerging professionals, this article was written to help leadership educators facilitate better within diverse training contexts. In it, I aim to help leadership educators increase their training confidence and participants’ experience during training. This article explores how to effectively create welcoming and courageous training environments for students of any identity. Although there have been recent attacks on diversity and inclusion professionals, roles, and departments, it does not negate that social identity and lived experiences influence student's real‐world application of leadership.

As a full‐time leadership studies lecturer and professional inclusive leadership speaker, I teach approximately 100 students across three core courses each semester and speak to thousands of people from around the world. I have taught leadership studies minor students at two doctoral universities (R2; American Council on Education, 2023). I have learned that students bring their identities to every level of schools, including elementary, secondary, and post‐secondary schools, whether schools are ready or not. When training across diverse contexts is done correctly, contemporary students want more. In this article, I will discuss how to create and maintain welcoming and courageous training environments by (1) building trust, (2) acknowledging social identity, (3) identity‐based storytelling, and (4) experiential learning activities.

## BUILDING TRUST

When leadership educators gain trust, it makes it easier for participants to reflect, engage, learn, and deal with conflict within diverse training contexts. Training across diverse contexts requires vulnerability without shame to build trust (Brown, 2018), which increases the capacity to cultivate belonging among group members of different social identities. Strayhorn (2012) insists trust, support, and building bonds are essential to educational success for all students. He further explains how the community can only exist once members feel a sense of belonging. The same can be said for creating welcoming and courageous training spaces for diverse student populations and training contexts; eadership learning can only happen once individuals feel a sense of belonging in the training space.

When training is welcoming and courageous, leadership educators can create space for forgiveness. I facilitate inclusive leadership training with thousands of students and professionals a year. In each session, I provide examples of relevant bias incidents to help participants see how these incidents happen and can have lasting impacts on people, communities, and cultures. After contextualizing the incidents, I ask every group, “If we shame, blame, guilt, or ostracize these individuals, do they get better at inclusion?” Not one person has said yes. Therefore, if oppressive behaviors are inescapable and inherent (Bell, 1992; Ladson‐Billings & Tate, 1995; Patton et al., 2015; Yosso, 2005), how must leadership educators create a welcoming environment for everyone, including those with opposing values? Leadership educators must not blame the participants; they must blame the system, allowing them to create a space for forgiveness in the leadership learning process.

The contextual landscape of higher education is changing. Therefore, if someone does or says something oppressive, leadership educators must use that opportunity to ask themselves or someone else where that came from. How does that belief help this community? How can that belief system impact the people in the room? As leadership educators begin their work with others, they must tell participants that oppressive behaviors are tied to their community's structures, institutions, and systems (Adams et al., 2007).

Once participants begin to look at these issues as systemic, they also start to share how their oppressive social identity experiences are connected to the systems they come from. The sharing opens the opportunity to create trust between participants because they are vulnerable to values that were placed on them by the community in which they grew up. Often, oppressive values are placed on participants years prior to training or education that broadens their perspective. Although these values no longer align with who they grew into, through a lens of social identity, sharing those stories is a powerful tool to increase trust. When participants of all social identities can openly share their experiences in training spaces, it promotes empathy and vulnerability, which creates trust.

Some participants shared how their social identity experiences impacted others, which is a powerful reflexive exercise of empathy and vulnerability. Once, after an inclusive leadership training, a professional told me that they were struggling to motivate one of their subordinates, who was much younger than them. She stated, “I just do not get it,” but my training helped her see that she was comparing her experiences from the 1900s to his experiences in 2024. She was living in the past, which is healthy when focusing on oneself, but that is not always true when working with others. She was not allowing him to define his experiences in the present, which may have triggered him to disconnect or distance himself from her.

Leadership educators must be able to see participants as individuals and collectives simultaneously. Through a lens of social identity, leadership educators need to create a space where participants belong, no matter the participant's social identity and experiences. Leadership educators can and must identify and establish a shared sense of identity to establish a welcoming and courageous environment. Since participants are individually and collectively connected to the physical training space, leadership educators can use geographic location, education, race, gender, careers, goals, learning styles, (dis)abilities, and more to establish a shared sense of identity. Binding participants across social identity creates belonging and contributes to a trusting environment.

Leadership educators need tools to gain trust and provide opportunities for participants to trust when facilitating training. Since there are multiple levels of understanding of individual and collective identities, training aims to collectively engage individuals in a process of social influence that achieves a shared outcome. The goal is to bind different backgrounds, experiences, and perspectives toward a shared outcome that motivates and inspires everyone to believe in everyone. Individuals will trust when the facilitation process influences participants to acknowledge themselves and others through a lens of diversity within and beyond the training context.

A pair‐and‐share or role‐playing activity can serve as an introduction for participants to connect and share something more profound about themselves. Another simple practice is to have participants discuss what their full name means and provide prompts that help them tell more profound stories about themselves. For example, an introductory activity called “What is in a name?” can be great for building trust. Each person needs to find a partner. Once everyone has a partner, they should be given prompts to answer about their name. Some prompts include telling a story about your name or what your name means. Where does your name come from? Who named you and why? What did you feel about your name as a kid, and how does that compare to now?

This activity forces participants to see the power of names and consider the power of trust building through sharing about names. In the debrief, participants can get into small or large groups to discuss the importance of names in building trust. Names provide a wide range of insight into a person's background, culture, social identity, and even personal identity. Mispronouncing names can inadvertently lead to a marginalized experience in training. This activity can be eye‐opening for participants and leadership educators because it reveals how unconscious bias, assumptions, and stereotypes can hinder creating welcoming environments across diverse identities.

## ACKNOWLEDGING THE IMPACTS OF SOCIAL IDENTITY

Leadership educators who infuse social identity into their facilitation create courageous spaces for diverse audiences to share truth and increase empathy for individuals with differing levels of privileged and marginalized experiences. Understanding the intersections of leadership behavior and social identity experiences should challenge leadership educators to identify how their identities impact individuals, groups, and communities within the training context. Since every training group is different, each attempt at facilitation should challenge the leadership educator to assess the necessary changes needed to center participants’ relationships, learning, behaviors, and experiences.

Leadership educators must consider how their identities influence their behaviors in relation to the identities of each participant. Facilitators must greet participants as they enter the space to create a welcoming environment, which may cause a need to engage in different styles of introductions based on the social identities of participants. To meet the training needs of all participants, leadership educators should understand their and the participants’ identity positionality (Kezar & Lester, 2010) when developing and enhancing leadership learning. Before creating welcoming spaces for others, trainers must experience welcoming spaces to infuse them in their pedagogy.

While attacks on diversity, equity, and inclusion (DEI) training have been on the rise (Chaisson‐Cardenas, 2022; Edwards, 2023; Harper et al., 2024; Hernández, 2024; Murray et al., 2023; Russell‐Brown, 2024; Watson, 2024; Zahneis, 2024), skills to facilitate cross‐cultural leadership learning experiences and dialogue about oppression have been decreasing. Increased guilt and shame‐based approaches to leadership training have the potential to cause more harm and decrease motivation to attend training that addresses diversity of any kind, making it more difficult for trainers to experience and implement diversity into their work. I learned to create welcoming spaces by volunteering to teach an intergroup dialogue (train‐the‐trainer) course for resident assistants. That experience caused me to deconstruct my identity journey to develop meaning‐making tools (Drath & Palus, 1994) to enhance welcoming and courageous spaces for students.

Since social identity is continuously in motion during facilitation, no matter the context, what may be welcoming for some groups may be uncomfortable for others. Leadership educators could start each facilitation by asking one or all three of these questions: (1) Who is in the room (name, major, class, age, gender pronoun, race, ethnic background, nationality, or others)? (2) Why are you here? and (3) What do you hope to gain by being here? This strategy helps leadership educators create welcoming and courageous training environments because it centers on why participants are present and the direction needed for the training to be valuable.

Connecting the leadership educator's identity to the context of the training space and the participant's goals is critical for creating welcoming and courageous training spaces. Social identity is one's sense of self in relation to others (Jones & Abes, 2013; Jones et al., 2016). Interconnecting participants' identities can positively impactcommunication, motivation, behavior, support, and comfort. To address issues of exclusion and to create a safe, positive training space, leadership educators need to increase empathy and vulnerability (Brown, 2018). Meaning‐making tools like stories, values, interests, goals, and other tools (Drath & Palus, 1994) help participants better understand their past and present and predict their future.

I never intended to become a storyteller or intergroup dialogue facilitator. However, these strategies transformed my practice by increasing my vulnerability and empathy. Increasing the vulnerability and empathy of leadership educators and participants within a diversity training space contributes to a welcoming and courageous environment. Although this article is not about shame, blame, or guilt, those feelings can instantly unravel training facilitation. Allowing participants to share commonalities, what they hope to gain, or gratitude promotes a shared sense of belonging.

## IDENTITY‐BASED STORYTELLING

As a qualitative researcher, I believe stories are the glue that binds communities, cultures, ancestry, and history. Stories are the ultimate guide toward belonging (Albert & Vadla, 2009; Armstrong, 2021; Auvinen et al., 2013). Facilitating leadership learning through a lens of social identity is a reflexive exercise (Mahoney, 2017; McCarron et al., 2023). Leadership educators must reflect on and practice sharing their stories often to become proficient at using them to influence diverse training contexts. Identity‐based storytelling positions leadership educator's lived experiences, behaviors, emotions, actions, and perceptions at the center of learning in the training space. Leadership influence is a relational process that promotes change by finding common ground between leadership educators and participants (Kroll, 2023). Telling relevant and inspirational stories binds the leadership educator's past to the participant's future, which increases the opportunity for connection.

According to Armstrong (2021), storytelling is: (1) an essential leadership skill and practice, (2) improves self‐awareness of identity, and (3) constructs a narrative and counter‐narratives of understanding organizations. Social identity stories are the best way to share positionality within diverse training contexts. Stories are a potent meaning‐making tool (Drath & Palus, 1994). When I do inclusive leadership training, participants are compelled to tell stories because I tell my story. The most powerful stories are the ones where individuals share about doing inclusion wrong, how they became more self‐aware, and how they redeemed themselves within the community. Although sharing stories helps create welcoming and courageous environments, it does not guarantee it. Stories must be relevant to maximize impact. Sharing relevant stories is the most critical factor for leadership educators to increase the likelihood of creating a welcoming and courageous environment. Relevant stories connect participants to the facilitation, facilitator, and contextual environment. They allow participants to reflect deeply, tap into their emotions and creativity, and reimagine their behaviors and practices (Armstrong, 2021).

Besides sharing relevant stories to create a welcoming and courageous environment, other elements must be included to ensure effectiveness. Identity‐based storytelling in diverse contexts often centers on oppressive experiences full of hurt and pain. To thwart hurt and pain from taking over the training space, leadership educators’ identity‐based stories must be relevant and understood by the audience, show courage and an ability to overcome, and conclude with a learning lesson that participants can use for leadership learning.

Understanding one's own experiences and connecting those to participants’ backgrounds creates opportunities for new connections. Injecting identity‐based stories gives life to culture, perspectives on culture, and participants' cultures within the training context. Identity‐based stories help bond participants to the contextual environment, leadership educator, and the training process. Identity‐based storytelling allows participants to evaluate their identity experiences and place themselves within the leadership educator's story as socio‐contextual actors who can change and create change. When participants begin sharing how they change and create change, it increases vulnerability, belonging, and courage.

One study found that stories influence leadership in six areas: (1) motivation, (2) inspiration, (3) defusing conflict, (4) influencing superiors, (5) discovering a focus, and (6) constructing trust (Auvinen et al., 2013). Identity‐based stories enable participants to see themselves in similar situations and gauge how they would influence others based on their social group memberships and the social group memberships of their communities. Identity‐based stories encourage participants to push their boundaries beyond their own lived experiences. The leadership educator's story sets the tone and direction of the participant's experience in a diverse training context. Identity‐based stories are critical to creating vulnerability because they are role models and build courage and authenticity with participants. Sharing identity‐based stories helps participants develop their authentic leadership voice (Albert & Vadla, 2009).

Leadership educators must also note that managing the nuances and impacts of participants’ stories during training takes intergroup dialogue skills and practice. Being a leadership educator does not make you a counselor; you must keep the audience's goals and training at the forefront of the participant's mind. When participants start sharing identity‐based stories, leadership educators must ensure that participants share stories they have overcome and can offer leadership learning for the group. Once leadership educators use their identity‐based story to set the tone and direction for the training, they should deepen the connections between participants by using activities.

## EXPERIENTIAL LEARNING ACTIVITIES

Training within diverse contexts today, across industries, has become lectures and webinars by leading scholars. Although lectures and webinars can be informative, they do not guarantee that participants internalize the information to create healthier leadership habits (Fujimoto & Härtel, 2017; Kroll, 2023). Lectures can inspire and increase emotions but rely solely on listening. Although listening is essential, it can exclude most of the participants who fall into other learning styles (Poon et al., 2000).

Experiential learning enables participants to engage in activities critically through their lens of cognition. Traditional lectures are known to be less effective than experiential learning activities because they are considered boring, unengaging, and passive. Formal lectures assume that learning is one‐dimensional and does not actively engage multiple learning styles and modalities (Poon et al., 2000). Experiential learning activities engage participants in numerous learning styles and modalities to construct new knowledge (Kroll, 2023). Experiential learning activities increase accessibility within diverse training contexts by improving participant's ability to critically examine individual and collective lived experiences, thoughts, emotions, and actions through their primary learning style.

Traditional approaches to educating within diverse training contexts are less likely to increase acceptance and courage because educating across diversity is complex. It takes years for many people to fully understand and articulate the most popular scholars, articles, books, theories, and models. It is ill‐advised to believe that a lecture will exponentially increase leadership learning or change leadership behavior in many participants. On the contrary, helping participants experience concepts has a better probability of increasing knowledge and belonging because it simultaneously addresses multiple learning styles. “The work” to maintain a welcoming and courageous environment throughout training is through experiential learning activities that include numerous learning styles, capabilities, and modalities.

Experiential learning is rooted in equity, compared to conventional learning, which is rooted in anti‐equity (Kroll, 2023). In conventional learning, trainers have a predetermined message to deliver to participants. The goal is for participants to absorb the message, making them the sole experts in the training space. This approach is problematic because it typically does not engage multiple learning styles and modalities. It also gives trainers absolute power over participants' learning, with little room for them to contribute. Experiential learning is the opposite.

Experiential learning is predicated on participants’ engagement with the leadership educator, other participants, and their leadership learning. It offers opportunities for learning to be shared, uncovered, and cultivated. Experiential learning includes the vision and voice of participants (Kroll, 2023). Including the voices and visions of participants increases the value of the training and those involved in it. When all participants are valued and can contribute, participants feel more welcomed and courageous. Also, experiential learning creates space for multiple learning styles and empowers participants to explore learning in ways that are most meaningful for them.

Including multiple learning styles during experiential learning activities helps leadership educators create, advance, and embed a sense of shared identity between participants. Including multiple learning styles increase participant satisfaction and the likelihood of leadership learning (Poon et al., 2000). Leadership educators must incorporate participants’ goals and desires into activities to increase leadership learning (Fujimoto & Härtel, 2017). Identifying participants’ learning styles and goals increases trust. When different voices and perspectives have equal opportunity to contribute to the direction and learning of the training, it maintains a welcoming and courageous learning environment.

When participants recognize that they will get what they need and that everyone needs something different, trust, empathy, and vulnerability increase (Brown, 2018). When participants have a voice and feel a shared sense of empowerment to discuss their values and experiences in training, they create a collective identity through their goals, desires, and aspirations. Experiential learning through self‐assessment activities allows leadership educators to facilitate an identity‐construction process to make connections to others. Leadership educators should aim to create a shared experience using leadership concepts and theories when training across diverse contexts. Allowing participants the opportunity to self‐select their primary leadership traits, styles, or behaviors (Northouse, 2021) helps them co‐construct the meaning of leadership concepts and identify their differences and similarities. Once participants decide on a specific characteristic that best identifies them, group them with others in a specific area in the training space. After everyone selects a group, elaborate on each characteristic with behaviors congruent with their selected group. Also, have them construct stories of how their primary characteristics intersect with their social identity experiences.

Intersecting leadership behaviors and social identity experiences with participants increase their critical thinking skills and self‐awareness. When participants agree with descriptions and tell their stories, it affirms that they are in the right training space and that leadership educators are effective and impactful during training. Once everyone has found a group that best identifies with their leadership characteristics, have them find a partner to share stories with and discuss strategies to maximize their leadership practice. The assessment and discussion combined make the training experiential (Kroll, 2023) and beneficial to participants developing a shared identity. When participants increase their self‐awareness and understanding of others, leadership educators increase the likelihood of maintaining a welcoming training environment from start to finish.

Experiential learning activities that unearth stories, leadership characteristics, and values from upbringing, backgrounds, education, race, gender, personality type, strengths, and preference all increase shared learning, identity, and goals in training. Experiential learning puts participants in the driver's seat of their behavioral change process. Giving participants agency and power to position and reposition themselves as a learning method increases the chance of maintaining a welcoming environment. While most conventional leadership training focuses on professional skill development, experiential learning activities within diverse training contexts focus on participants’ history, current behaviors, and positive behavioral changes.

## CONCLUSION

Leadership educators aim to effectively lead and manage diverse training contexts to produce positive change in and for individuals, groups, communities, societies, and organizations. For positive change to happen in training participants, leadership educators must increase participants’ self‐awareness and vulnerability. This article outlined four strategies that leadership educators can implement to build and maintain a welcoming and courageous environment throughout training that produces leadership learning outcomes for all participants, regardless of their social identity. Leadership educators must have experience in this area to train participants across diverse contexts.

Although having a training framework is critical, experiencing training helps leadership educators practice dialoguing about social issues within a training context.

Experiencing a welcoming training environment also exposes leadership educators to many strategies to train within diverse contexts. The strategies I provided are influenced by years of learning from experts who train. The Program on Intergroup Relations at the University of Michigan and the Social Justice Training Institute are role models for me, which helped me understand what it meant to analyze and contextualize privileged and marginalized experiences to elevate leadership learning through an equity and inclusion lens. I aimed to help students process and increase their relationship‐building, collaboration, and positive social action skills, and in turn, students helped me do the same.

## References

[yd20646-bib-0001] Adams, M. , Bell, L. A. , & Griffin, P. (Eds.). (2007). Teaching for diversity and social justice (2nd ed.). Routledge. https://psycnet.apa.org/doi/10.4324/9780203940822

[yd20646-bib-0002] Albert, J. F. , & Vadla, K. (2009). Authentic leadership development in the classroom: A narrative approach. Journal of Leadership Education, 8(1), 72–92. 10.12806/v8/i1/ab2

[yd20646-bib-0003] American Council on Education . (2023, November 17). 2025 research designations. Carnegie Classification of Institutions of Higher Education. https://carnegieclassifications.acenet.edu/carnegie‐classification/research‐designations/

[yd20646-bib-0004] Armstrong, J. P. (2021). Guest editor's introduction: Storytelling and leadership. Journal of Leadership Studies, 14(4), 45–49. 10.1002/jls.21727

[yd20646-bib-0005] Auvinen, T. , Aaltio, I. , & Blomqvist, K. (2013). Constructing leadership by storytelling—The meaning of trust and narratives. Leadership & Organization Development Journal, 34(6), 496–514. 10.1108/lodj-10-2011-0102

[yd20646-bib-0006] Bell, D. A. (1992). Faces at the bottom of the well: The permanence of racism. Basic Books.

[yd20646-bib-0007] Bertrand Jones, T. , Guthrie, K. L. , & Osteen, L. (2016). Critical domains of culturally relevant leadership learning: A call to transform leadership programs. New Directions for Student Leadership, 152, 9–21. 10.1002/yd.20205 27870522

[yd20646-bib-0008] Brown, B. (2018). Dare to lead: Brave work, tough conversations, whole hearts. Random House.

[yd20646-bib-0009] Chaisson‐Cardenas, J. (2022). *Resistance and backlash from the perspective of PK‐12 educational diversity, equity, and inclusion professionals (DEIP) in a predominantly White Midwestern state* (Publication No. 29169903) [Doctoral dissertation], The University of Iowa. ProQuest Dissertations & Theses Global.

[yd20646-bib-0010] de Bray, C. , Musu, L. , McFarland, J. , Wilkinson‐Flicker, S. , Diliberti, M. , Zhang, A. , Branstetter, C. , & Wang, X. (2019). Status and trends in the education of racial and ethnic groups 2018. National Center for Education Statistics. https://nces.ed.gov/pubsearch/pubsinfo.asp?pubid=2019038

[yd20646-bib-0011] Drath, W. H. , & Palus, C. J. (1994). Making common sense: Leadership as meaning‐making in a community of practice: Leadership as meaning‐making in a community of practice. Center for Creative Leadership.

[yd20646-bib-0012] Dugan, J. P. (2017). Leadership theory: Cultivating critical perspectives. John Wiley & Sons.

[yd20646-bib-0013] Edwards, O. J. (2023). Experiences of Black chief diversity officers at predominantly White institutions during the anti‐critical race theory movement. (Publication No. 30314124) [Doctoral dissertation], Florida State University. ProQuest Dissertations & Theses Global.

[yd20646-bib-0014] Fujimoto, Y. , & Härtel, C. E. J. (2017). Organizational diversity learning framework: Going beyond diversity training programs. Personnel Review, 46(6), 1120–1141. 10.1108/PR-09-2015-0254

[yd20646-bib-0015] Harbin, B. (2016). Teaching beyond the gender binary in the university classroom. Updated by Roberts, L. M. et al. (2020). Vanderbilt Center for Teaching. Retrieved September 10, 2024, from https://cft.vanderbilt.edu/guides‐sub‐pages/teaching‐beyond‐the‐gender‐binary‐in‐the‐university‐classroom/

[yd20646-bib-0016] Harper, S. , Chang, M. J. , Cole, E. R. , Patton, L. , Garces, M. , & Smith, S. M. (2024). Truths about DEI on college campuses: Evidence‐based expert responses to politicized misinformation. Diverse: Issues in Higher Education. https://race.usc.edu/2024/03/19/2517/

[yd20646-bib-0017] Haslam, S. A. , Steffens, N. K. , Peters, K. , Boyce, R. A. , Mallett, C. J. , & Fransen, K. (2017). A social identity approach to leadership development: The 5R program. Journal of Personnel Psychology, 16(3), 113–124. 10.1027/1866-5888/a000176

[yd20646-bib-0018] Hernández, T. K. (2024). Can CRT save DEI: Workplace diversity, equity & inclusion in the shadow of anti‐affirmative action. UCLA Law Review https://www.uclalawreview.org/can‐crt‐save‐dei/

[yd20646-bib-0019] Jones, S. R. , & Abes, E. S. (2013). Identity development of college students: Advancing frameworks for multiple dimensions of identity. John Wiley & Sons, Incorporated.

[yd20646-bib-0020] Kezar, A. , & Lester, J. (2010). Breaking the barriers of essentialism in leadership research: Positionality as a promising approach. Feminist Formations, 22(1), 163–185. 10.1353/nwsa.0.0121

[yd20646-bib-0021] Kroll, J. R. (2023). Preparing leadership educators: A comprehensive guide to theories, practices, and facilitation skills (1st ed.). Stylus Publishing, LLC.

[yd20646-bib-0022] Ladson‐Billings, G. , & Tate, W. F. (1995). Toward a critical race theory of education. Teachers College Record, 97(1), 47–68. 10.1177/016146819509700104

[yd20646-bib-0023] Mahoney, A. D. (2017). Being at the heart of the matter: Culturally relevant leadership learning, emotions, and storytelling. Journal of Leadership Studies, 11(3), 55–60. 10.1002/jls.21546

[yd20646-bib-0024] McCarron, G. P. , McKenzie, B. L. , & Yamanaka, A. (2023). Leadership identity development. New Directions for Student Leadership, 2023(178), 31–43. 10.1002/yd.20552 37309856

[yd20646-bib-0025] Murray, T. A. , Oerther, S. , & Simmons, K. J. (2023). Anti‐DEI legislation targeting colleges and universities: Its potential impacts on nursing education and the pursuit of health equity. Nursing Outlook, 71(4), 101994. 10.1016/j.outlook.2023.101994 37336170

[yd20646-bib-0026] Northouse, P. G. (2021). Introduction to leadership: Concepts and practice (5th ed.). Sage Publications, Inc.

[yd20646-bib-0027] Passel, J. S. , & Cohn, D. (2008). U.S. population projections: 2005–2050. Pew Research Center. http://www.pewresearch.org/

[yd20646-bib-0028] Patton, L. D. , Harper, S. R. , & Harris, J. (2015). Using critical race theory to (re)interpret widely studied topics related to students in US higher education. In A. M. Martinez‐Aleman , B. Pusser , & E. M. Bensimon (Eds.), Critical approaches to the study of higher education: A practical introduction (pp. 193–219). Johns Hopkins University Press.

[yd20646-bib-0029] Parker, K. (2021, November 8). What's behind the growing gap between men and women in college completion? Pew Research Center. https://www.pewresearch.org/shortreads/2021/11/08/whats‐behind‐the‐growing‐gap‐between‐men‐and‐women‐in‐college‐completion/

[yd20646-bib-0030] Poon, J. M. L. , Stevens, C. K. , & Gannon, M. J. (2000). Effects of training method and learning style on cross‐cultural training outcomes. Research and Practice in Human Resource Management, 8(2), 73–97.

[yd20646-bib-0031] Russell‐Brown, K. (2024). The multitudinous racial harms caused by Florida's stop woke and anti‐DEI legislation. University of Florida Levin College of Law Legal Studies, 24(11), 786–839. https://papers.ssrn.com/sol3/papers.cfm?abstract_id=4573301

[yd20646-bib-0032] Schwartz, N. (2023, October 30). Fall 2023 enrollment trends in 5 charts. Higher Ed Dive. https://www.highereddive.com/news/fall‐2023‐enrollment‐trends‐5‐charts/697999/

[yd20646-bib-0033] Strayhorn, T. L. (2012). College students' sense of belonging: A key to educational success for all students (1st ed.). Routledge. 10.4324/9780203118924

[yd20646-bib-0034] Watson, L. (2024, February 14). Anti‐DEI efforts are the latest attack on racial equity and free speech: ACLU. American Civil Liberties Union. https://www.aclu.org/news/free‐speech/anti‐dei‐efforts‐are‐the‐latest‐attack‐on‐racial‐equity‐and‐free‐speech

[yd20646-bib-0035] Yosso, T. J. (2005). Whose culture has capital? A critical race theory discussion of community cultural wealth. Race, Ethnicity and Education, 8(1), 69–91. 10.1080/1361332052000341006

[yd20646-bib-0036] Zahneis, M. (2024, January 31). Diversity offices, statements, and training are banned in Utah's public colleges. The Chronicle of Higher Education. https://www.chronicle.com/article/diversity‐offices‐statements‐and‐training‐are‐banned‐in‐utahs‐public‐colleges

